# Time-reversal-breaking induced quantum spin Hall effect

**DOI:** 10.1038/srep43049

**Published:** 2017-02-21

**Authors:** Wei Luo, D. X. Shao, Ming-Xun Deng, W. Y. Deng, L. Sheng

**Affiliations:** 1 National Laboratory of Solid State Microstructures and Department of Physics, Nanjing University, Nanjing 210093, China; 2 Collaborative Innovation Center of Advanced Microstructures, Nanjing University, Nanjing 210093, China; 3 School of Science, Jiangxi University of Science and Technology, Ganzhou 341000, China

## Abstract

We show that quantum spin Hall (QSH) effect does not occur in a square lattice model due to cancellation of the intrinsic spin-orbit coupling coming from different hopping paths. However, we show that QSH effect can be induced by the presence of staggered magnetic fluxes alternating directions square by square. When the resulting Peierls phase takes a special value 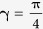, the system has a composite symmetry Θ*Ρ*_−_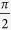 with Θ the time-reversal operator and *Ρ*_−_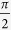 transforming the Peierls phase from γ to γ − 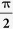, which protects the gapless edge states. Once the phase deviates from 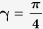, the edge states open a gap, as the composite symmetry is broken. We further investigate the effect of a Zeeman field on the QSH state, and find that the edge states remain gapless for 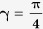. This indicates that the QSH effect is immune to the magnetic perturbation.

The quantum spin Hall (QSH) effect is a new quantum state of matter, which originates from spin-orbit coupling (SOC) and has attracted much attention in recent years because of its fundamental interest and potential applications in spintronic devices. The QSH effect was first theoretically predicted independently by Kane and Mele^1^ and by Bernevig and Zhang^2^. Soon after, the QSH effect was observed experimentally in HgTe quantum wells^3^, following theoretical prediction^4^. The discovery of the QSH effect has inspired theoretical proposals for topological insulators in three dimensions^5^,^6^,^7^, which have also been confirmed experimentally^8^,^9^. The QSH systems are two-dimensional (2D) topological insulators protected by time-reversal (TR) symmetry, whose edge states are robust against perturbations of nonmagnetic disorder. Therefore, the currents carried by the edge states are dissipationless and immune to nonmagnetic scattering. A key ingredient of the QSH effect is a strong intrinsic SOC, which acts as spin-dependent magnetic fluxes coupled to the electron momenta.

In the model proposed by Kane and Mele^1^, the intrinsic SOC in graphene would open a band gap in the bulk and also establish spin-filtered edge states that traverse the band gap, giving rise to the QSH effect. Even though the Kane-Mele model is hardly achievable, because the intrinsic SOC in graphene is too weak to produce an observable effect under realistic conditions^10^,^11^, it captures the essential physics of the QSH state with nontrivial band topology^12^,^13^. In the presence of spin conservation, the two spin sectors of a QSH system behave like two independent quantum Hall (QH) systems without Landau levels^14^. When the electron Fermi level is inside the bulk band gap, they contribute opposite quantized Hall conductivities, so that the total Hall conductivity vanishes but the spin Hall conductivity is quantized. When the spin conservation is destroyed by the Rashba SOC, the edge transport can remain to be dissipationless^1^,^15^ due to the TR symmetry. In this case, a QSH system can no longer be divided into two QH subsystems, and the existence of the gapless edge states has been attributed to the nontrivial topological properties of bulk energy bands, which can be characterized by a nonzero Z_2_ index^16^ or a nonzero spin Chern number^17^,^18^.

The TR symmetry was often considered to be a prerequisite for the QSH effect. In a TR invariant QSH system, the two oppositely moving edge states are connected to each other under TR, and so have opposite spin orientations. As a result, elastic backscattering from nonmagnetic disorder is forbidden. However, it was found that even when the TR symmetry is broken, the bulk topological properties of a QSH system remain intact until the bulk energy gap closes^19^. In this case, there usually appears a small gap in the energy spectrum of the edge states, but not always. For example, for a sufficiently smooth confining potential, the edge states can be gapless, at the expense of gap opening in the spin spectrum near the edges^20^. Another example is that when a narrow ferromagnetic region is created through magnetic doping near the edge of a QSH sample, which induces spatial separation of the two helical edge states, the edge states can become gapless so that the QSH effect is stabilized^21^.

In this paper, we consider a square lattice with staggered magnetic fluxes with directions alternating square by square. The staggered magnetic fluxes break the TR symmetry, and induce an accompanying Peierls phase factor in the electron hopping elements. We will show that the phase factor leads to a nonzero intrinsic SOC and provides the possibility for the QSH effect. When the phase factor takes a special value 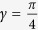, the system has a composite symmetry 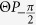, with Θ the time-reversal operator and 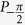 a gauge transformation that transforms the Peierls phase from γ to 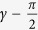. Under this symmetry, the two dispersion branches of the edge states at the same edge cross each other at the 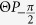-invariant points. When the phase factor deviates from 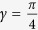, the system may be still in the QSH state, but as the composite symmetry is broken, there will be a small energy gap in the edge states just like the case in the TR-symmetry-broken QSH state^19^. We further investigate the effect of a Zeeman field on the QSH state, which violates the 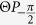 symmetry no matter what value the Peierls phase γ takes. Remarkably, it is found that the edge states in the QSH phase remain to be gapless, if the Peierls phase takes the special value 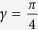. We have not found a certain explicit symmetry to protect the gapless edge states, different from what is usual in low dimensions^22^. Similar to the case without the Zeeman field, once the Peierls phase deviates from 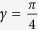, the edge states open a gap.

## Results

### Model Hamiltonian

**Figure 1:**
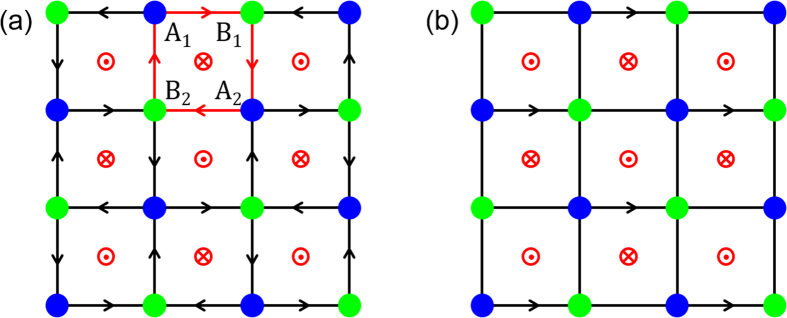
Schematic of the lattices. The blue and green filled circles represent the lattice sites of sublattices *A* and *B*; the red dots and crosses in the circles represent opposite magnetic fluxes and the arrows represent the Peierls phases of hopping *γ*. (**a**) and (**b**) correspond to different gauges.

We consider the model as shown in Fig. 1(a). On a square lattice, there exist magnetic fluxes with directions alternating square by square. The magnetic fluxes induce an accompanying Peierls phase factor *γ* in the hopping elements along the arrow^23^. Due to the existence of the magnetic fluxes, the translation symmetry is broken, and the lattice is divided into two sublattices denoted by *A* and *B*. The lattice constant is taken to be *d* = 1 in the following calculations. For such a two-dimension compound lattice, the primitive lattice vectors can be chosen to be **a**_**1**_ = (1, 1) and **a**_**2**_ = (1, −1). The corresponding primitive reciprocal lattice vectors are **b**_**1**_ = (*π, π*) and **b**_**2**_ = (*π*, −*π*).

The Hamiltonian of the system under consideration is given by


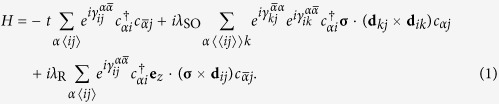


Here, 
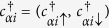
 is the electron creation operator on site *i* with *α* = *A, B* standing for *A, B* sublattices. The first term is the usual nearest-neighbor hopping term with 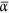 being the sublattice different from *α* and the angular bracket in 〈*ij*〉 standing for nearest-neighbor sites. The second term is the intrinsic SOC with coupling strength *λ*_SO_, where ***σ*** is the spin Pauli matrix vector, *i* and *j* are two next nearest-neighbor sites, *k* are their common nearest-neighbor sites, and vector **d**_*ik*_ points from *k* to *i*. The last term stands for the Rashba SOC with coupling strength *λ*_R_. In addition, a Peierls phase factor appears in all of the hopping terms due to the staggered magnetic fluxes. Here, we adopt a symmetric gauge, and the Peierls phase satisfies 
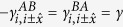
, 
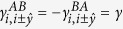
. The Peierls phase will be confined in the range 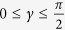.

It is clear that when the Peierls phase γ is nonzero, the TR symmetry is broken. As we will show below, it is the TR symmetry breaking that induces the QSH effect in the present case of a square lattice. Different from the honeycomb lattice of the Kane-Mele model, in which there exists only one common nearest neighbor between a pair of next nearest neighbor sites, there are two common nearest neighbors between a pair of next nearest neighbor sites in the square lattice. Thus, in the intrinsic SOC term, there exist two hopping paths connecting a pair of next nearest neighbor sites, and the total intrinsic SOC is the sum of them. If the Peierls phase is zero, the intrinsic SOC vanishes, as the cross products **d**_*kj*_ × **d**_*ik*_ from the two paths cancel. Take *A*_1_, *A*_2_ in Fig. 1(a) for example. The intrinsic SOC, coming from the sum of the two paths *A*_2_ → *B*_1_ → *A*_1_, *A*_2_ → *B*_2_ → *A*_1_, can be expressed as 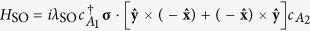, where 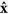 and 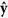 represent the unit vectors in the x and y directions, respectively. It is clear that the sum in the square brackets is zero. However, the situation is different in the presence of a Peierls phase of hopping. Taking a nonzero Peierls phase into account, we have


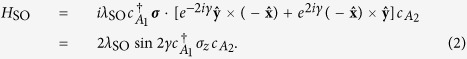


One can see that only if the Peierls phase *γ* ≠ 0 or 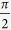, there exists a nonzero intrinsic SOC, which is crucial for inducing the QSH effect.

### Edge states

By making a Fourier transformation, we obtain the Hamiltonian in momentum space as


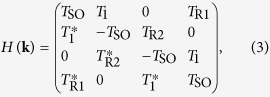


where the base vectors are chosen as {c_*A*↑_, c_*B*↑_, c_*A*↓_, c_*B*↓_}, and


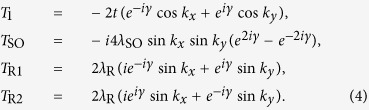


Solving the energy eigenequation and by some algebra, it is found that for 
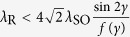
 with 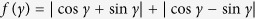, there is a finite energy gap of magnitude 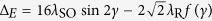 between the conduction and valence bands. For 
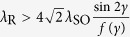
, the energy gap vanishes, and the conduction and valence bands cross at points 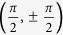 in the momentum space.

Due to the existence of the nonzero intrinsic SOC, the system is possible in the QSH state, when the band gap is open. First, it is clear that the system is in the QSH state when *λ*_R_ = 0. In this case, the electron spin is conserved, and the system can be divided into two spin sectors. As long as *γ* does not take the value 0 or *π*/2, the contributions to the intrinsic SOC from different paths do not cancel, and act as spin-dependent magnetic fluxes coupled to the electron momentum. The two spin sectors of the QSH system behave like two independent QH systems without Laudau levels. When *λ*_R_ ≠ 0, the spin conservation is destroyed, the system can no longer be divided into two QH systems, and unconventional topological invariants, the Z_2_ index or spin Chern numbers are needed to classify the system. Here, we use the spin Chern numbers to characterize the topological quantum phase. Employing the standard method^18^,^19^ to calculate the spin Chern numbers, we obtain C_±_ = ±1 for 
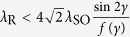
, which corresponds to a topological QSH insulator.

**Figure 2:**
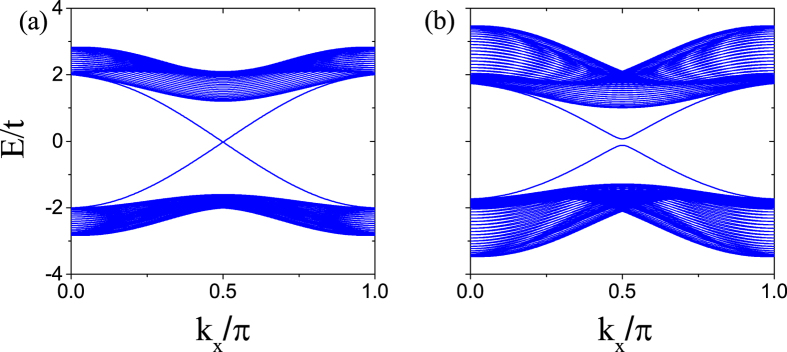
Energy spectrum versus wave vector *k*_*x*_ for a strip geometry for (**a**) 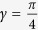 and (**b**) 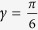. The parameters are chosen as *λ*_SO_ = 0.2*t*, and *λ*_R_ = 0.1*t*.

According to bulk-edge correspondence, the spin Chern numbers C_±_ = ±1 indicate that there are a pair of edge modes counterpropagating at the open boundary of the system, which are spin polarized. It is worth pointing out that for the Kane-Mele model, when a Zeeman field, a TR-symmetry-breaking term, is present, there appears a small gap in the spectrum of the counter-propagating edge states due to the mixing of the two edge states on the same boundary. In the present model, the TR symmetry is also broken due to the staggered magnetic fluxes, so it is necessary to examine the behavior of edge states. To study the spectrum of the edge states, we consider an infinitely long strip geometry running along the *x* direction with width (*N*_*y*_ − 1)*d*. Open boundary conditions are imposed at the two edges of the strip. The system has translational invariance along the *x* direction, so that the x component of the momentum *k*_*x*_ is a good quantum number. The energy spectra corresponding to the QSH phase are shown in Fig. 2(a) and (b) for different Peierls phases 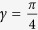 and 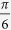, respectively. One can notice a significant distinction between the edge states displayed in the two figures. In the case of 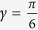, as shown in Fig. 2(b), it is apparent that there is a energy gap in the edge modes. This is to be expected, because the TR symmetry is broken, and the two edge states on the same boundary are mixed. However, for 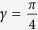, the edge states are gapless. The two edge modes at the same sample edge cross each other at 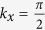, as shown in Fig. 2(a). In general, in low dimensions, the band degeneracy is protected by some symmetry. For example, in the Kane-Mele model, the gapless edge states are protected by the TR symmetry. In the present case, there should be certain symmetry in the system to protect the gapless nature of the edge states for 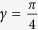. We will search such a symmetry in the next section.

### Symmetry protection of the gapless edge states

In order to search the symmetry, we first examine the characteristics of the edge states localized near one boundary. Considering the energy-momentum relation and the spin polarizations of the edge states, we have 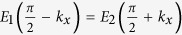 and 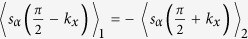, where *α* = *x, y, z*, and the subscript is the edge state index. Owing to 
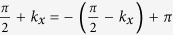
 with *π* being the reciprocal lattice vector of the strip geometry, one can find that this is similar to Kramers’ theorem of TR. Therefore, we conjecture that the symmetry protecting the gapless edge states consists of the TR. In addition, the fact that the edge states are gapless only when 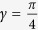, indicates that the symmetry contains a factor closely relating to 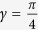. We find the corresponding symmetry operator to be 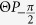, where 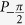 is a operator that transforms the accompanying phase in the hopping coefficients *γ* to 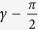, and Θ = *i*σ_*y*_*K* represents the TR operator for spin-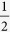 fermions, with *K* being the complex conjugation operator. For 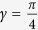, the model is invariant under the composite transformation 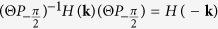.

A Bloch function of the electrons has the form 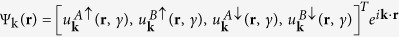. The symmetry operator Θ*P*_− *π*/2_ acts on the Bloch function as follows


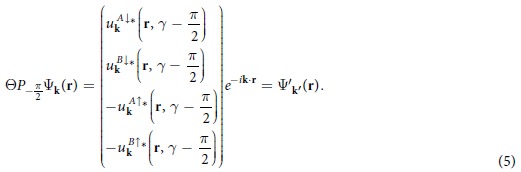


Because 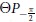 is the symmetry operator of the system, Ψ′_**k′**_(**r**) must be a Bloch wave function of the system. Thus, we obtain **k′** = −**k**.

From Eq. (5), it is easy to show that the operator 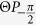 has the effect to transform electron wave vector as **k** → −**k** = **k′**. If **k′** = **k** + **K**, where **K** is the reciprocal lattice vector, then **k** is a 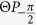-invariant point in momentum space. If **G** is a 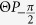-invariant point, then we have 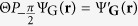. Due to 
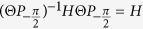
, Ψ_**G**_(**r**) and Ψ′_**G**_(**r**) are both the eigenstates of Hamiltonian *H* and have the same eigenenergy *E*(**G**). In order to further understand the role of 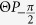, we study the effect of 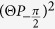 on the Bloch function Ψ_**G**_(**r**) based on Eq. (5), and find


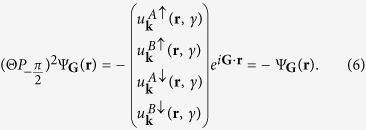


We should note that when the second operator 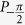 acts on Ψ′_**G**_(**r**), which transforms the accompanying phase in the hopping coefficients *γ*′ to 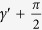 rather than 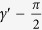 because the TR reverses the direction of the magnetic fluxes. Finally, we obtain 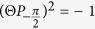 at all the 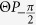-invariant points. It is known that if a system is invariant under the action of an antiunitary operator and the square of the operator is not equal to 1, there must be degeneracy protected by this antiunitary operator. Thus, the bands must be degenerate at the 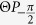-invariant points in the Brillouin zone. For a strip geometry, just the *k*_*x*_ is a good quantum number, so there exist only two 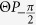-invariant points *k*_*x*_ = 0 and 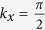. At one given edge, there are two dispersion branches cross each other at the 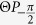-invariant point 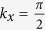. However, at *k*_*x*_ = 0 they merge into the bulk and pair with the edge states of the other boundary.

From the above discussion, one can see that the symmetry 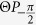 is essentially a TR. The factor 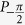 is a consequence of the symmetric gauge that we have chosen. Due to the freedom of gauge transformation, for the staggered magnetic fluxes shown in Fig. 1(a), we can choose different gauges, namely, the accompanying phases in the hopping terms can be chosen in different manners. This causes the Hamiltonian to be in different forms, and have different symmetry operators. If we choose a proper gauge, the factor 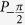 in the symmetry operator can be removed. Specifically, if we choose the gauge shown in Fig. 1(b), it is clear that when the accompanying phase 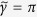, which corresponds to 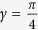 in Fig. 1(a), the symmetry of the system will be characterized by the ordinary TR operator.

### The effect of a Zeeman field

**Figure 3:**
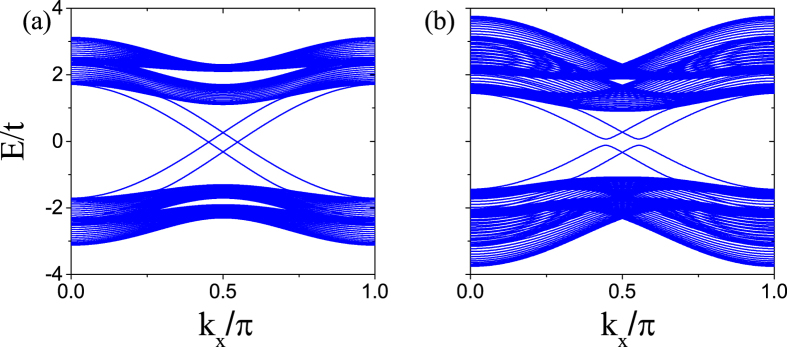
Energy spectrum versus wave vector *k*_*x*_ for a strip geometry for (**a**) 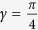 and (**b**) 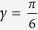 in the presence of Zeeman field. The parameters are chosen as *g* = 0.2*t, λ*_SO_ = 0.2*t*, and *λ*_R_ = 0.1*t*.

It is well known that QSH effect is not stable to magnetic perturbations, which break the TR symmetry. In the Kane-Mele model, a uniform Zeeman field will gap out the edge states of the QSH phase. Now we also consider the effect of a Zeeman field with strength *g* in the present system. The Hamiltonian of the Zeeman field is given by 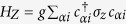. It is clear that the Zeeman field *H*_*Z*_ violates the symmetry 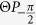, no matter what value the accompanying phase *γ* takes. The energy spectrum for the strip geometry with 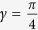 is plotted in Fig. 3(a). To our surprise, when the system is in the QSH phase with C_±_ = ±1, even if g ≠ 0, the edge states are gapless as long as 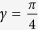. One can easily see that the Zeeman field does not lift the degeneracy of the two edge states localized near a given boundary but shifts the position of the degenerate point through raising the energy of one edge state and reducing the energy of the other edge state. This means that the Zeeman field does not mix the two edge states, so does not cause backscattering of the edge states, and the transport in the edge states is still dissipationless at zero temperature. Similarly to the case of *g* = 0, however, once the accompanying phase *γ* deviates from 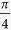, the edge states open a small gap as shown in Fig. 3(b).

As discussed above, the degeneracy in low dimensions is usually protected by certain symmetry. However, in the presence of the Zeeman field, we have not found the symmetry protecting the gapless edge states. Similarly, we calculate the spin polarizations of the edge states, and find that the relations 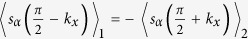 no longer hold. Through numeric calculation, it is found that the degenerate points of the edge states localized near the two boundaries approximately satisfy 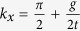, 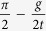. The degenerate points are not at high-symmetry points, and shift with changing the strength of the Zeeman field. Because the edge states and degenerate points can no longer provide any useful clue, it is difficult to find the hidden symmetry.

Lastly, we make a comparison with previous works, refs 23 and 24, which also describe the robust QSH phase under TR symmetry breaking. In both the works, the symmetry protecting the gapless edge states is Θ*T*_1/2_, with Θ the TR, and *T*_1/2_ a primitive-lattice translation. Owing to the primitive-lattice translation, the edge states protected by Θ*T*_1/2_ are no longer robust against nonmagnetic or magnetic disorder. In contrast, in our work, the symmetry protecting the gapless edge states does not depend on the primitive-lattice translation, so the edge states are robust against nonmagnetic disorder. More interestingly, the fact that the edge states in our model remain gapless in the presence of a Zeeman field and spin-flipping term indicates that the edge states are also robust against magnetic disorder.

## Conclusion

We have investigated the QSH effect in a square lattice with staggered magnetic fluxes alternating directions square by square, which lead to a nonzero intrinsic SOC through inducing an accompanying phase in the hopping elements. When the phase factor takes a special value 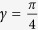, the system has a composite symmetry 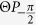, which protects the degeneracy of edge states at the 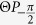-invariant point 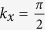. Once the phase deviates from 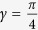, the edge states open a gap, as the composite symmetry is broken. More interestingly, the system is stable in the presence of a Zeeman field. As long as 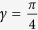, the edge states keep being gapless. This means that magnetic perturbation does not mix the two edge states localized at the same edge, so do not cause backscattering of the edge states. We believe that in the presence of the Zeeman field, the gapless edge states are still protected by certain hidden symmetry, although we have not found it. Similarly, once the accompanying phase deviates from 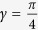, the edge modes open an energy gap.

## Additional Information

**How to cite this article**: Luo, W. *et al*. Time-reversal-breaking induced quantum spin Hall effect. *Sci. Rep.* **7**, 43049; doi: 10.1038/srep43049 (2017).

**Publisher's note:** Springer Nature remains neutral with regard to jurisdictional claims in published maps and institutional affiliations.
